# A disease modification drug screening platform targeting neuronal hyperexcitability and seizures in Alzheimer’s disease‐associated mouse models

**DOI:** 10.1002/alz.086241

**Published:** 2025-01-09

**Authors:** Kevin M Knox, Leanne M Lehmann, Aaron Del Pozo Sanz, Larissa Robinson‐Cooper, Stephanie Davidson, Melissa Barker‐Haliski

**Affiliations:** ^1^ University of Washington, Seattle, WA USA

## Abstract

**Background:**

Early‐onset Alzheimer’s disease (EOAD) associated with amyloid precursor protein (APP) duplications or presenilin (PSEN) variants increases risk of seizures. Targeting epileptiform activity with antiseizure medicine (ASM) administration to AD patients may beneficially attenuate cognitive decline (Vossel et al, *JAMA Neurology* 2021). However, whether mechanistically distinct ASMs differentially suppress seizures in discrete EOAD models is understudied (Lehmann et al, *Neurochem Res* 2021). To fill this gap, we validated a moderate‐throughput drug screening program consisting of well‐established ASM discovery tests applied in three discrete EOAD‐associated models: PSEN2 knockout (KO), PSEN2‐N141I (Volga German variant), and APP^swe^/PS1^dE9^ mice. Critically, PSEN2‐N141I model does not demonstrate accumulated amyloid beta (Ab) whereas APP/PS1 mice show increased Ab expression by ∼6 months‐old. We hypothesized EOAD‐associated models would differentially influence focal seizure threshold, susceptibility to evoked focal seizures, and ASM sensitivity with advanced age.

**Method:**

PSEN2 KO, PSEN2‐N141I, APP/PS1, and wild‐type (WT) mice were tested beginning at 2‐months‐old and up to 14‐months‐old in acute and chronic seizure screens: minimal clonic and 6 Hz focal seizure threshold, 6 Hz electroconvulsive test, corneal kindling, and kainic acid‐induced status epilepticus (KA‐SE), consistent with our published methods for ASM discovery (Barker‐Haliski et al, *Neurochem Res* 2017; Knox et al, *Epilepsia* 2021). Mice were assessed in two seizure threshold tests every 2 months until 12‐months‐old. Mechanistically distinct ASMs (diazepam, valproic acid, lamotrigine, and levetiracetam) profiled genotype‐specific pharmacosensitivity in the seizure assays (6 Hz, corneal kindling, and KA‐SE).

**Result:**

Functional connectivity differences appeared as notable blunting of age‐related increases in 6 Hz seizure threshold of APP/PS1 mice by as early as 4‐months‐old (APP/PS1 median convulsant current: 24.6 mA [22.2‐26.9] versus WT: 31.7 [27.8‐36.9]). Chronic kindled seizure susceptibility varied by genotype, with ASM‐specific differences in seizure control between EOAD models. Specifically, kindled seizures of aged APP/PS1 mice were more sensitive to valproic acid and levetiracetam, whereas valproic acid and lamotrigine were more potent in aged PSEN2‐N141I mice.

**Conclusion:**

EOAD‐associated risk genes do not equally influence age‐related seizure susceptibility or impact ASM efficacy; seizures in AD may require precision medicine interventions. Further ASM profiling focused on attenuating neuronal hyperexcitability may uncover novel disease‐modifying strategies for AD.

